# Coral-associated viral communities show high levels of diversity and host auxiliary functions

**DOI:** 10.7717/peerj.4054

**Published:** 2017-11-17

**Authors:** Karen D. Weynberg, Patrick W. Laffy, Elisha M. Wood-Charlson, Dmitrij Turaev, Thomas Rattei, Nicole S. Webster, Madeleine J.H. van Oppen

**Affiliations:** 1 Australian Institute of Marine Science, Townsville, Queensland, Australia; 2 School of Chemistry and Molecular Biosciences, University of Queensland, Brisbane, Queensland, Australia; 3 Department of Microbiology and Ecosystem Science, Division of Computational Systems Biology, University of Vienna, Vienna, Austria; 4 Australian Centre for Ecogenomics, University of Queensland, Brisbane, Queensland, Australia; 5 School of Biosciences, University of Melbourne, Melbourne, Victoria, Australia

**Keywords:** Virus, Coral, *Symbiodinium*, Metagenomics, Holobiont, GBR

## Abstract

Stony corals (Scleractinia) are marine invertebrates that form the foundation and framework upon which tropical reefs are built. The coral animal associates with a diverse microbiome comprised of dinoflagellate algae and other protists, bacteria, archaea, fungi and viruses. Using a metagenomics approach, we analysed the DNA and RNA viral assemblages of seven coral species from the central Great Barrier Reef (GBR), demonstrating that tailed bacteriophages of the *Caudovirales* dominate across all species examined, and ssDNA viruses, notably the *Microviridae*, are also prevalent. Most sequences with matches to eukaryotic viruses were assigned to six viral families, including four Nucleocytoplasmic Large DNA Viruses (NCLDVs) families: *Iridoviridae, Phycodnaviridae, Mimiviridae, and Poxviridae*, as well as *Retroviridae* and *Polydnaviridae*. Contrary to previous findings, *Herpesvirales* were rare in these GBR corals. Sequences of a ssRNA virus with similarities to the dinornavirus, *Heterocapsa circularisquama* ssRNA virus of the *Alvernaviridae* that infects free-living dinoflagellates, were observed in three coral species. We also detected viruses previously undescribed from the coral holobiont, including a virus that targets fungi associated with the coral species *Acropora tenuis*. Functional analysis of the assembled contigs indicated a high prevalence of latency-associated genes in the coral-associated viral assemblages, several host-derived auxiliary metabolic genes (AMGs) for photosynthesis (*psbA*, *psbD* genes encoding the photosystem II D1 and D2 proteins respectively), as well as potential nematocyst toxins and antioxidants (genes encoding green fluorescent-like chromoprotein). This study expands the currently limited knowledge on coral-associated viruses by characterising viral composition and function across seven GBR coral species.

## Introduction

Reef-building corals are keystone taxa of coral reefs; they are responsible for the deposition of a three-dimensional calcium-carbonate framework that constitutes the reef, and are the ecosystem’s main primary producers (Muscatine, Mccloskey & Marian, 1981). The coral animal lives in close association with a diverse suite of macroscopic and microscopic symbionts, and is thus a complex holobiont (Rohwer et al., 2002). A growing body of evidence supports a critical role for microbial symbionts in the coral holobiont (Blackall, Wilson & Oppen, 2015; Bourne, Morrow & Webster, 2016). *Symbiodinium* spp. are obligate symbionts and meet most of the nutritional requirements of the coral host animal by translocating photosynthate (fixed carbon) and nitrogen to the coral tissues (Muscatine, Mccloskey & Marian, 1981; Muscatine & Weis, 1992). Bacterial symbionts play a role in nitrogen fixation (Lema, Willis & Bourne, 2012; Lesser et al., 2004), sulphur metabolism (Raina et al., 2009), and coral holobiont immune responses (Krediet et al., 2013), while some bacteria can become opportunistic pathogens under certain conditions (Harvell et al., 2007; Weynberg et al., in press). The diversity and functional roles of other groups of symbionts within these holobiont communities are poorly studied. This is particularly true for viruses, i.e., the eukaryotic viruses, archaeal viruses, and bacteriophages, whose identity and diversity is still largely undescribed for many coral species. Earlier reports of viruses associated with corals were based on transmission electron microscopy observations of virus-like particles within coral tissue or present in the coral surface mucus layer (Davy & Patten, 2007; Wilson et al., 2005). More recently, metagenomics has been used to identify taxonomic groups of viruses present in certain coral species from different geographical locations and exposed to different environmental stressors (Dinsdale et al., 2008; Laffy et al., 2016; Thurber et al., 2008; Weynberg et al., 2014). However, our knowledge of the identity of most viral communities and their function within corals is still scant. Several dsDNA viral families have been reported from stony corals including: *Phycodnaviridae; Mimiviridae*; *Poxvirirdae*; *Iridoviridae*; *Herpesviridae*; *Ascoviridae;* with *Caudovirales* or tailed bacteriophages being the most dominant order (reviewed in Thurber et al., 2017). However, while previous studies have assessed the taxonomic composition of DNA viruses in corals, insights into RNA virus assemblages and viral functional genes are scarce. Here we utilize the recently developed computational pipeline HoloVir (Laffy et al., 2016) to describe the viral taxonomic diversity and gene function based on DNA and RNA viromes of seven scleractinian coral species and their surrounding seawater from the central GBR.

## Materials and Methods

### Sample collection, processing, and sequencing

Five species of scleractinian coral—*Acropora tenuis, Fungia fungites, Goniastrea aspera, Galaxea fascicularis* and *Pocillopora verrucosa*—were collected in early March 2013, under the Great Barrier Reef Marine Park Authority collection permit number G15/37272.1, from sites around Orpheus Island Research Station (OIRS) in the inshore central GBR. Fragments (∼10 cm diameter) were collected from three colonies, or three single-polyped corals in the case of *F. fungites,* and were returned to the lab’s flow-through seawater tanks until processing (within 3–4 h of collection). Processing was conducted as described in (Weynberg et al., 2014), including tissue removal via air-blasting, mechanical disruption using bead-beating, cesium chloride (CsCl) density fractionation to purify virus fractions, and buffer exchange to remove cesium salts prior to DNA and RNA extraction. This method ensures viral capsids remain intact prior to nucleic acid extraction following nuclease treatment. Amplification of viral DNA and RNA nucleic acids was performed using RP-SISPA, a sequence-independent amplification step (Weynberg et al., 2014), and sequenced using Nextera XT MiSeq 250 bp paired-end sequencing (Illumina, Hayward, CA, USA) at the Ramaciotti Centre, University of New South Wales, Sydney, Australia.

Two additional species of *Pocillopora* (fragments of approximately 10 cm diameter from three colonies) were included in this study. *P. damicornis* was collected at Trunk Reef in late November 2012, and *P. acuta* was collected at Davies Reef in early October 2013, both mid-shelf reefs in the central GBR ∼120 km from Orpheus Island. *Pocillopora* species were identified in the field based on colony morphology, and species ID was confirmed with a genetic assay (Torda et al., 2013). All species except *P. acuta* were collected in triplicate (i.e., three colonies) and pooled prior to sequencing. *P. acuta* samples were not pooled prior to sequencing. A seawater sample (20 L) was collected in the vicinity of the *P. acuta* samples to compare the holobiont and seawater virus communities. Seawater was transported to the Australian Institute of Marine Science (AIMS) for immediate filtration (0.22 µm Sterivex PES) and FeCl flocculation. In brief, 0.5 mL of FeCl (10 g/L stock) was added to 5 L of seawater, shaken for 1 min (repeated every 20 min), and stored at 25 °C for 1 hr. Flocculated viral particles were collected on a fresh Sterivex (Durapore PDVF), and ∼2 mL 0.2 M ascorbate-0.1 M EDTA-Mg buffer (made day of, *pH* = 6.0) were added to saturate the filter. The filter unit was capped with Parafilm, shaken vigorously to begin the dissociation of viral particles, and stored at 4 °C until the filter appeared clear. The solution was then purged from the filter, and the recovered viral particles were loaded onto a CsCl gradient and processed as described in (Weynberg et al., 2014).

### Sequence analysis

Virome data sets were analysed following the HoloVir protocol (Laffy et al., 2016), an analysis protocol specifically designed for taxonomic and functional analysis of holobiont-associated virus communities. HoloVir utilizes a two-tiered analysis approach, performing taxonomic and marker gene analysis to identify viral community composition, as well as the detection of any potential contamination issues, together with a Swiss-Prot keyword enrichment analysis to characterise the viromic functional profiles.

### Quality control, sample dereplication and single read analysis

Sequencing data sets underwent quality trimming using FastQC (version 1.11.5) (Andrews, 2010) overlapping reads were identified and merged using PEAR (Zhang et al., 2014) (Unmerged reads were fused together, separated by 10 padding n residues. Merged reads were dereplicated using cd-hit-est (Li & Godzik, 2006) using a global identity threshold of 99%. Merged and non-merged reads were combined prior to single read BLAST+ analysis for taxonomic assignment, and single read analysis was used to validate assembled gene analysis.

### Contig assembly and gene prediction

De novo assembly of viral sequences was performed using CLC Genomics workbench 8.5.1 with subsequent filtering steps for a minimum of 3× coverage and a minimum contig length of 1,000 bp for DNA viromes and 500 bp for RNA viromes. Gene prediction was performed on the resulting contigs using MetaGeneAnnotator (Noguchi, Taniguchi & Itoh, 2008). The resulting predicted genes were subsequently subjected to taxonomic assignment, marker analysis and functional assessment. Since the *P. acuta* samples were not pooled prior to sequencing, a single sequence run was used to maintain read number in further analyses.

### Taxonomic assignment

Comparison of predicted genes to the viral RefSeq database (Brister et al., 2015) was performed via BLAST sequence similarity searches using default parameters (Altschul et al., 1990). Taxonomic assignment was subsequently performed using MEGAN5 Last Common Ancestor (LCA) default parameters, using a minimum support parameter of five matches for taxonomic assignment (Huson et al., 2011). A cellular and viral marker database was generated as described in Laffy et al. (2016) and used in sequence similarity comparisons (via BLAST with default parameters, and MEGAN5 LCA default parameters with a minimum support parameter of one match for taxonomic assignment) to confirm viral RefSeq taxonomic assignments and identify potential cellular contaminants. Taxonomic assignment was defined at the species level, and the 60 most abundant taxonomic assignments were determined across all samples and visualized using ggplot in R.

### Functional analysis

The function of predicted genes from virome assemblies was determined by performing BLAST sequence similarity searches to the UniprotKB/Swiss-Prot database (UniProt Consortium, 2015), with an e-value cutoff of 10^−10^. Swiss-Prot keywords were then assigned to predicted genes based on the best hit. Identified Swiss-Prot keywords were collated for each viral metagenome, and the frequency of each keyword was calculated, relative to the proportion of that keyword within the SwissProt database. The 50 most abundant Swiss-Prot keywords were identified across all samples and visualized using ggplot in R.

## Results and Discussion

This study characterised the viral diversity across seven coral species of the central GBR and provided taxonomic and functional information with an additional comparison of viral communities that exist in seawater sampled in the GBR. Analysis of DNA and RNA derived coral viromes revealed a broad taxonomic diversity of viruses associated with central GBR corals. For the coral samples, ∼8.10^5^–3.10^6^ raw reads were obtained for each of the DNA and RNA viromes, and ∼3.10^5^ and ∼7.10^5^ reads were obtained from the water sample (Table 1). The number of assembled predicted genes was highly variable, ranging from ∼100–44,000 per sample (Table 1), with generally lower numbers for RNA compared to the DNA viromes. The viromes contained representatives from 23 dsDNA families (including six of the ten families that collectively form the nucleocytoplasmic large dsDNA viruses, or NCLDVs), six ssDNA families, one dsRNA virus, two ssRNA families and three retrotranscribing viral families. Many viral genes involved in latency and viral host infection, viral replication, propagation and particle assembly were observed, particularly in the DNA viromes. Data sets generated from these samples were submitted to Genbank Sequence Read Archive (see Table 1 for accession numbers).

**Table 1:** Sequencing statistics of seven coral holobiont viral communities. Sequencing statistics of DNA and RNA viromes associated with coral holobiont communities of seven Great Barrier Reef coral species.

Host species	Template	Accession number (Bioproject # PRJNA302344)	#raw reads	#contigs	N50	Longest contig	#predicted genes
*Acropora tenuis*	DNA	SAMN02709831	1,620,989	4,268	1,530	13,669	10,679
*Fungia fungites*	DNA	SAMN04272222	1,605,748	778	1,365	7,098	1,779
*Goniastria aspera*	DNA	SAMN04274802	1,170,146	15,173	1,614	48,259	44,198
*Galaxea fascicularis*	DNA	SAMN04274801	1,869,983	989	1,501	5,386	1,512
*Pocillopora acuta*	DNA	SAMN04277425	2,213,465	18,029	1,615	40,341	33,726
*Pocillopora damicornis*	DNA	SAMN02709826	2,659,198	2,188	1,617	20,529	5,651
*Pocillopora verrucosa*	DNA	SAMN04277423	9,328,233	10,351	1,594	67,301	29,381
Seawater	DNA	SAMN06849075	320,701	1,006	1,491	18,804	3,116
*Acropora tenuis*	RNA	SAMN02709832	853,227	1,517	731	5,386	1,499
*Fungia fungites*	RNA	SAMN04274763	1,323,170	354	693	1,597	377
*Goneastrea aspera*	RNA	SAMN04274806	1,337,144	3,953	727	5,693	5,144
*Galaxea fascicularis*	RNA	SAMN04277306	1,796,476	934	679	5,386	921
*Pocillopora acuta*	RNA	SAMN04277426	1,445,938	3,761	928	7,499	4,389
*Pocillopora verrucosa*	RNA	SAMN04277424	2,928,367	67	739	1,462	106
Seawater	RNA	SAMN06849076	667,528	60	699	1,795	102

### Taxonomic assignments

#### Viruses that target prokaryotes

**Figure 1:**
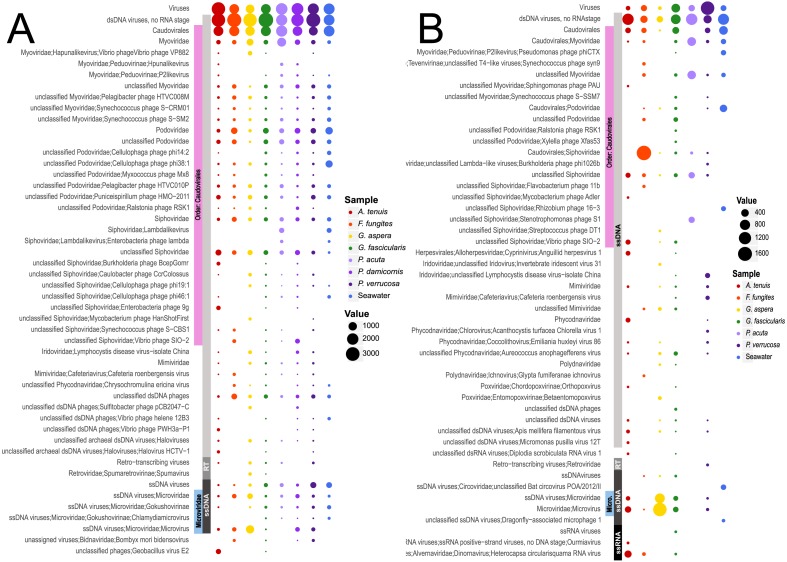
Taxonomic analysis of viruses from seven GBR coral species and surrounding seawater. Taxonomic analysis of viruses from seven coral species and surrounding seawater sampled on the GBR. Relative abundance of the 50 most abundant DNA (A) and RNA (B) viral taxa based on viral Refseq BLASTx comparisons to predicted genes from assigned assembled contigs. Abundances were adjusted based on contig coverage values for each virome community. MEGAN5 default LCA parameters were used to assign taxonomy.

The metavirome sequence data sets were dominated by viruses of prokaryotes, consistent with previous studies in other marine invertebrates (reviewed in Thurber et al., 2017). The most abundant viruses in the DNA viromes from all seven coral species were the dsDNA bacteriophages in the order *Caudovirales* (Figs. 1A and 1B), reflecting the high abundance and diversity of bacterial hosts within the coral holobiont (∼10^7^ prokaryotic cells/cm^2^ of coral surface area; 10^2^–10^4^ OTUs per coral colony (Blackall, Wilson & Oppen, 2015)). Previous coral microbial and viral metagenome studies on other species or from other locations also found high abundances of *Caudovirales* (Correa et al., 2016; Littman, Willis & Bourne, 2011; Soffer, Zaneveld & Vega Thurber, 2014; Wegley et al., 2007). Their prevalence in the RNA viromes was unexpected as viral particles were DNase-treated prior to viral genome isolation. In the DNA viromes, *Caudovirales*-like sequences were relatively evenly divided across the three families *Myoviridae*, *Siphoviridae* and *Podoviridae* in all seven coral species and the seawater (Fig. 1A). *Siphoviridae* contain many prophage types (King et al., 2011) (Fig. 1A), indicating a likely prevalence of lysogenic viruses in the coral holobiont. This is consistent with a high proportion of latency genes identified in the functional analyses (see section *Functional annotation of coral viromes* below).

An average 6% and 36% (DNA and RNA viromes, respectively) of all predicted viral genes were most similar to ssDNA viruses, in particular phages in the family *Microviridae*. *Microviridae* have previously been reported as an abundant component of coral metaviromes, possibly because the polymerase used in multi-displacement amplification (MDA, *phi-29*) preferentially amplifies small circular genomes (Correa et al., 2016; Littman, Willis & Bourne, 2011; Soffer, Zaneveld & Vega Thurber, 2014; Wegley et al., 2007). Our SISPA methods are template-independent PCR amplified, validating the abundance of *Microviridae* in corals. In this study, *Microviridae* were primarily represented by the genus *Microvirus,* which commonly infects enterobacteria, and three genera of the subfamily *Gokushnovirinae,* which are known to infect obligate intracellular parasitic bacteria such as *Bdellovibrio* and *Chlamydia* (King et al., 2011). These viruses may infect intracellular parasitic members of the *Halobacteriovorax* that have been confirmed to be present in the coral microbiome (Welsh et al., 2016), although this requires further experimental validation.

The DNA viromes of all but one coral species (*F. fungites*) contained sequences affiliated with the archaeal dsDNA virus, Halovirus. Haloviruses target archaea in the genus *Haloarcula* which are known to associate with corals (Thurber et al., 2009). Archaea are not as widespread as coral-associated bacteria (Wegley et al., 2007), but they can reach densities of >10^7^ cells per cm^2^ of coral surface area (Wegley et al., 2004). The presence of lemon-shaped virus-like particles, a common morphology of these viruses, in the surface mucus layer of corals (Davy & Patten, 2007) is also consistent with our viral sequence data.

#### Viruses that target eukaryotes

Of the total number of assembled contigs, between 0.5% (*F. fungites*) to 18% (*P. acuta*) of the DNA viromes matched eukaryotic viruses. The majority of predicted dsDNA eukaryotic viruses in the DNA viromes were assigned to five viral families, namely four NCLDV families: *Iridoviridae, Phycodnaviridae, Mimiviridae, Poxviridae*, as well as *Polydnaviridae* (Fig. 1). The NCLDVs *Ascoviridae* and *Marseilleviridae* were rare, as were other viral families (<0.5%; Fig. 1). The most predominant Iridovirus-like sequences had highest sequence similarity to lymphocystivirus, which commonly infect fish species (King et al., 2011) and have previously been detected in the Caribbean coral *Porites* sp. (Wegley et al., 2007). Mimivirus-like hits were detected in all coral species but not seawater and these findings are consistent with previous virome, transcriptome and TEM data showing the presence of *Mimiviridae* in *Acropora aspera* from the southern GBR (Correa et al., 2016), as well as in Caribbean and Hawaiian corals (Correa, Welsh & Thurber, 2013; Thurber et al., 2008). *Phycodnaviridae* are also known to be prevalent members of coral viral assemblages (Thurber & Correa, 2011). The presence of phycodnaviruses in corals is likely linked to the dinoflagellate endosymbiont (*Symbiodinium* spp.) or possibly to endolithic algae present in the coral skeleton (Verbruggen & Tribollet, 2011), as phycodnaviruses are known to infect algae (King et al., 2011). A recent study showed that NCLDV sequences with similarities to *Phycodnaviridae* and *Mimiviridae* were abundant in the transcriptomes of GBR *Symbiodinium* type C1 cultures (Levin et al., 2017), confirming members of these diverse viral families target *Symbiodinium*. These viral transcripts were regulated during experimental heat stress, suggesting they play a role in the *Symbiodinium* thermal stress response. Most recently, a transcriptomic study of viruses associated with *Symbiodinium microadriaticum* clade A1, revealed a prevalence of viruses related to the *Potyviridae*, and high expression of viral genes under heat shock treatment (Brüwer et al., 2017).

Contrary to previous reports on coral virus assemblages (Correa et al., 2016; Thurber et al., 2008), sequences related to the *Herpesviridae* were absent or present in very low abundance (<0.1% of all eukaryotic virus reads in five of seven of coral species sampled). Such low representation of herpesviruses may reflect differences in the abundance of these viruses across coral species, geographic locations and coral physiological states, but may also be due to the different computational approaches applied (Wood-Charlson et al., 2015).

Members of the *Retroviridae* were also seen in all coral species sequenced with the exception of *F. fungites.* Retro-transcribing viruses have previously been reported in corals (Correa et al., 2016; Correa, Welsh & Vega Thurber, 2012; Weynberg et al., 2014; Wood-Charlson et al., 2015) and are known to be abundant in diverse eukaryotes (Koonin, Dolja & Krupovic, 2015). In contrast, prokaryotes are predominantly infected by dsDNA bacteriophages with no reported retroviruses infecting bacteria or archaea. This suggests that eukaryotic members of the coral holobiont are the retroviral targets.

Three of the coral species (*A. tenuis, F. fungites* and *G. fascicularis*) contained sequences with similarity to a dinornavirus, *Heterocapsa circularisquama* ssRNA virus of the *Alvernaviridae,* that infects free-living dinoflagellates (Tomaru et al., 2004). This virus is likely a new divergent ssRNA virus that targets the coral algal symbiont, *Symbiodinium* (Levin et al., 2017). Gene transcripts of the major capsid protein were previously shown to be highly expressed in a thermo-sensitive *Symbiodinium* C1 population at ambient temperature, but not in a conspecific thermo-tolerant population under the same conditions, suggesting it may play a role in thermal susceptibility of this dinoflagellate coral endosymbiont (Levin et al., 2017). The presence of *Alvernaviridae* sequences in some, but not all, *Symbiodinium* transcriptome data sets (Correa, Welsh & Thurber, 2013; Levin et al., 2017) suggests not all *Symbiodinium* types and populations are infected with this virus.

**Figure 2:**
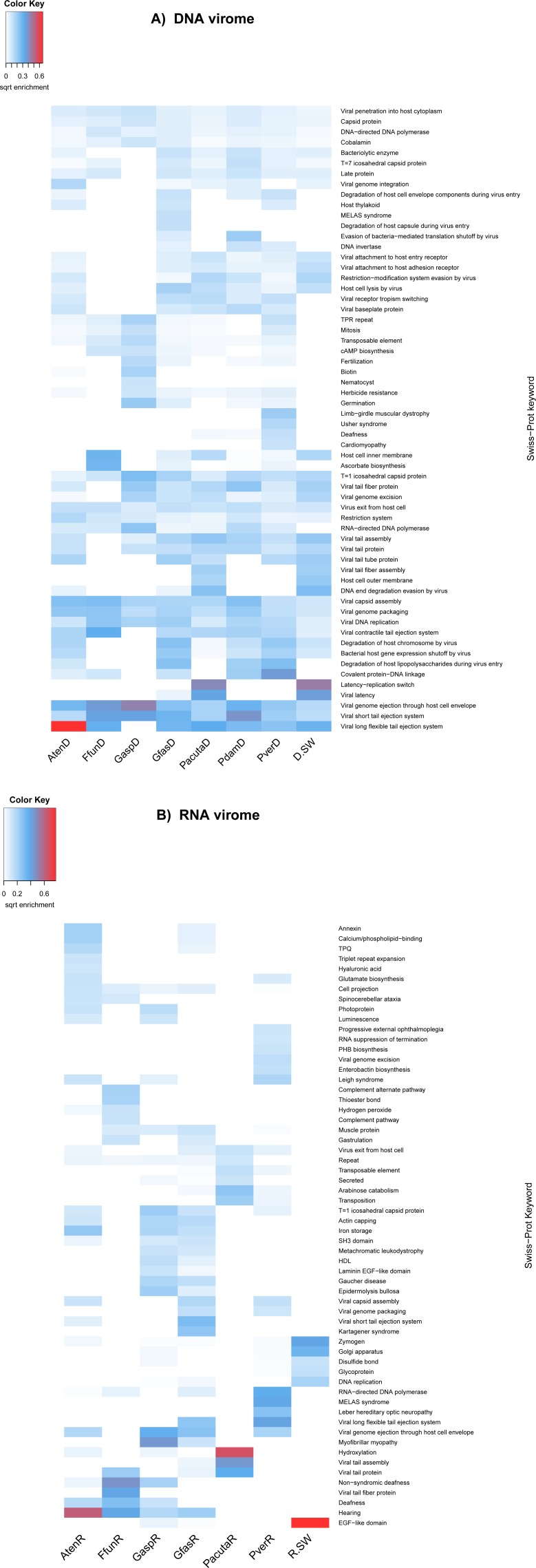
Predicted gene functional analysis of DNA (A) and RNA (B) viromes in seven GBR coral species and surrounding seawater. Functional enrichment based on the 60 most enriched Swissprot keywords from SwissProt BLASTx comparisons to assembled predicted genes. Keyword counts were adjusted based on contig coverage values for each virome community.

Sequences with similarities to *Bombyx mori* bidensovirus, a ssDNA virus belonging to the *Bidnaviridae* family (King et al., 2011; Krupovic & Koonin, 2014) were detected in four of the seven species but not in the surrounding seawater (Fig. 1A). A second viral group previously undescribed from corals, but detected in the RNA viromes of *A. tenuis* and *G. fascicularis,* had sequence similarity to the dsRNA mycovirus, *Diplodia scrobiculata* virus, which is known to infect endophytic fungi in terrestrial plants (King et al., 2011). Fungi are known to associate with the coral skeleton (Amend, Barshis & Oliver, 2012; Bentis, Kaufman & Golubic, 2000), and we hypothesize they may act as a host in corals. As dsRNA does not exist in cells during normal replication processes, this nucleic acid form can be exceptionally vulnerable to attack by nucleases and is less stable than DNA (Mertens, 2004), which may explain the absence of these viruses in previously reported coral viromes.

#### Functional annotation of coral viromes

The most abundant genes in the coral and seawater DNA viromes related to viral functions (Fig. 2A). ‘Latency-replication switch’ was the most common Swiss-Prot keyword, and ‘viral latency’ was also highly represented in *P.acuta* (and seawater), suggesting that prophages are a common feature in the holobiont of this particular coral species. This is consistent with the high prevalence of *Siphoviridae, Myoviridae* and *Podoviridae* observed in the taxonomic analyses. It is possible that the high prevalence of latency related genes may have originated from bacteriophages entering a lysogenic stage within their holobiont host genomes. In line with the predominance of bacteriophages in the taxonomic analyses, the second most abundant keyword was ‘viral long flexible tail ejection system’, which represents a viral protein that constitutes the noncontractile ejection system carried by some long-tailed prokaryotic viruses such as the *Siphoviridae*. Of the top 60 Swiss-Prot keywords, 17 fell in non-viral categories, eleven of which were absent from the seawater sample.

Several viral genes with host auxiliary function that may be beneficial to the host were detected. Viruses associated with three of the seven coral species examined (*A. tenuis*, *G. fascicularis* and *P. verrucosa*) had genes encoding proteins associated with the Swiss-Prot keyword ‘host thylakoid’. The thylakoid is the compartment within the chloroplast where light-dependent reactions of photosynthesis occur. The genes in Swiss-Prot associated with this keyword encode the photosystem II (PSII) D2 protein (*psbD*), a protein that, together with the D1 protein, forms the photochemically active reaction center of PSII (Barber, Chapman & Telfer, 1987). All seven coral species harboured genes of the key word ‘herbicide resistance’, which is represented as *psbA* encoding the PSII D1 protein. Some of these sequences matched the *psbA* gene from other dinoflagellates. Host genes encoding the D1 and D2 proteins have previously been observed in cyanophages that infect marine *Synechococcus* and *Prochlorococcus* (Lindell et al., 2004; Mann et al., 2003; Weigele et al., 2007), including those from coral atolls in the Line Islands (Dinsdale et al., 2008; Sharon et al., 2009). Impairment of PSII results in an increase in reactive oxygen species in the cell and may lead to coral bleaching (Warner, Fitt & Schmidt, 1996; Warner, Fitt & Schmidt, 1999). Therefore, viruses carrying PSII genes may alleviate and/or delay some of the damage to the *Symbiodinium* PSII from high seawater temperature, herbicides or other stressors, thereby providing energy and more time for their own replication.

The keywords ‘photoprotein’ and ‘luminescence’ were present in the *A. tenuis* and *G. aspera* RNA viromes. Both are represented by genes encoding green fluorescent-like protein (GFP) chromoproteins (CPs). GFP-like fluorescent proteins are responsible for the bright coloration many corals display, and are encoded by a large numbers of genes (Alieva et al., 2008). While the function of many fluorescent proteins remains ambiguous (Roth, 2014), there is some evidence for an antioxidant role of CPs (Palmer, Modi & Mydlarz, 2009). As viral infections are known to cause an increase in ROS in the infected cells (Bidle & Vardi, 2011), it is possible that these virally encoded genes may prolong the life of the coral host cells allowing the virus to produce more offspring than it would have without providing antioxidants to its host, although further experimental work would be needed to validate this mechanism.

Genes encoding proteins classified under the key word ‘nematocyst’ were highly abundant in one of the coral species sampled, *P. verrucosa*, and also present in *G. aspera*. These sequences match Delta-thalatoxin genes known from anemones (Oshiro et al., 2004). We speculate that these toxin genes acquired by coral-associated viruses may provide a benefit to the coral host by assisting in prey acquisition or repelling predators.

## Conclusions

Metagenomic analyses of viral assemblages from seven GBR coral species have revealed considerable viral diversity in corals. The abundance and diversity of bacteriophages suggests they target and control bacterial populations associated with corals. The presence of viral genes associated with functions such as nematocyst toxins and auxiliary metabolic processes indicate a potential benefit of viral infection for hosts within the holobiont, providing a foundation for further experimental investigation and validation.

##  Supplemental Information

10.7717/peerj.4054/supp-1 Supplemental Information 1 Statistical summary of seven GBR coral species DNA and RNA metavirome data

## Additional Information and Declarations

### Field Study Permissions

The following information was supplied relating to field study approvals (i.e., approving body and any reference numbers): Field permit G15/37272.1 was issued by the Great Barrier Reef Marine Park Authority.

### DNA Deposition

The following information was supplied regarding the deposition of DNA sequences: The DNA and RNA viromes described here are accessible via Genbank under Bioproject # PRJNA302344 and accession numbers SAMN02709831, SAMN04272222, SAMN04274802, SAMN04274801, SAMN04277425, SAMN02709826, SAMN04277423, SAMN06849075, SAMN02709832, SAMN04274763, SAMN04274806, SAMN04277306, SAMN04277426, SAMN04277424, and SAMN06849076.
