# Supplementary material for: Coral-associated viral communities show high levels of diversity and host auxiliary functions
Source: PeerJ. 2017 Nov 17;5:e4054. doi: 10.7717/peerj.4054 (PMC5695250; doi:10.7717/peerj.4054)
Supplement: Supplemental Information 1 [file peerj-05-4054-s001.docx]

**Table S1. Sequence data, contig assembly and gene prediction summary of seven* GBR coral species DNA and RNA metavirome data.**

| **Coral species virome** | **Raw read count** | **QC dereplicated read count** | **Contig count** | **N50** | **Longest contig** | **Number of bases assembled** | **Number of predicted genes** | **% cellular contamination** |
| --- | --- | --- | --- | --- | --- | --- | --- | --- |
| **DNA** |  |  |  |  |  |  |  |  |
| *Acropora tenuis* | 1,620,989 | 839692 | 4,268 | 1,530 | 13,669 | 6,709,451 | 10,679 | 0.4 |
| *Fungia fungites* | 1,605,748 | 906266 | 778 | 1,365 | 7,098 | 1,103,405 | 1,779 | 0 |
| *Galaxea fascicularis* | 1,170,146 | 842212 | 15,173 | 1,614 | 48,259 | 25,124,566 | 44,198 | 4.2 |
| *Goniastrea aspera* | 1,869,983 | 1130457 | 989 | 1,501 | 5,386 | 1,508,260 | 1,512 | 1.24 |
| *Pocillopora verrucosa* | 2,213,465 | 1663135 | 18,029 | 1,615 | 40,341 | 29,210,941 | 33,726 | 0.85 |
| *Pocillopora acuta* | 2,659,198 | 1483997 | 2,188 | 1,617 | 20,529 | 3,507,398 | 5,651 | 0.26 |
| *Pocillopora damicornis* | 9,328,233 | 6045130 | 10,351 | 1,594 | 67,301 | 16,967,610 | 29,381 | 5.5 |
| Seawater | 320,701 | 295125 | 1,006 | 1,491 | 18,804 | 1,608,447 | 3,116 | 0 |
| **RNA** |  |  |  |  |  |  |  |  |
| *Acropora tenuis* | 853,227 | 335510 | 1,517 | 731 | 5,386 | 1,142,142 | 1,499 | 2.9 |
| *Fungia fungites* | 1,323,170 | 259868 | 354 | 693 | 1,597 | 245,167 | 377 | 1.8 |
| *Galaxea fascicularis* | 1,337,144 | 464523 | 3,953 | 727 | 5,693 | 2,934,423 | 5,144 | 1.6 |
| *Goniastrea aspera* | 1,796,476 | 319580 | 934 | 679 | 5,386 | 658,076 | 921 | 2.1 |
| *Pocillopora verrucosa* | 1,445,938 | 847086 | 3,761 | 928 | 7,499 | 3,445,217 | 4,389 | 0 |
| *Pocillopora acuta* | 2,928,367 | 512847 | 67 | 739 | 1,462 | 50,637 | 106 | 2.2 |
| Seawater | 667,528 | 193969 | 60 | 699 | 1,795 | 42,490 | 102 | 0 |

*No RNA virome data available for *Pocillopora damicornis*
